# Research progress of artificial intelligence and machine learning in pulmonary embolism

**DOI:** 10.3389/fmed.2025.1577559

**Published:** 2025-03-27

**Authors:** Yue Li, Limin Zhang, Haoran Liu, Yanxia Li, Zhuo Liu

**Affiliations:** ^1^Department of Pulmonary and Critical Care Medicine, First Affiliated Hospital of Dalian Medical University, Dalian, China; ^2^Dalian Medical University, Dalian, China

**Keywords:** artificial intelligence, machine learning, pulmonary embolism, prediction, diagnosis, prognosis

## Abstract

The pathophysiology and clinical manifestations of pulmonary embolism are complex, heterogeneous, and the disease burden is severe, and its prediction and diagnosis are of major challenges. Artificial intelligence (AI) is a field of computer science that involves the development of programs and complex data analysis designed to replicate human cognitive processes. In recent years, with the continuous development of medical information technology, the application of AI in the diagnosis and treatment of diseases has made rapid progress, especially in the field of pulmonary embolism, which is mainly based on imaging. In this review, we summarize the current application prospects and directions of AI in early prediction, screening, diagnosis, and prognosis of PE, and discuss the main challenges and future of AI in pulmonary embolism (PE), in order to provide a theoretical basis for the application of AI in the risk assessment and standardized management of PE.

## 1 Introduction

Acute PE is the third leading cause of cardiovascular death worldwide (1), presenting with mild clinical symptoms or atypical features, and current diagnosis relies primarily on CT imaging, with computed tomography pulmonary angiography (CTPA) considered the diagnostic benchmark (2). However, interpreting CTPA results requires experienced medical expertise and avoiding missed and misdiagnosed due to subjective bias. Studies have shown that CTPA has a missed diagnosis rate of 14% and an overdiagnosis rate of 10% (3). Due to the insidious onset and diverse etiologies of PE, the detection rate is less than 5% (4), and the short-term mortality rate of untreated PE is as high as 30% (5, 6). Improving the survival rate of PE patients depends on early and accurate prediction, as well as active PE management. Therefore, early prediction, identification, and diagnosis of PE play a key role in patient outcomes (7, 8).

AI excels in medical image analysis as a subtype of information technology that uses algorithms to analyze (receive, process, and interpret) medical information and perform complex mathematical calculations (9). Machine learning (ML) is an emerging branch of artificial intelligence, whose core competitiveness lies in the ability to efficiently extract and analyze rich clinical features from massive case record databases, and accurately identify statistically significant patterns. Deep learning (DL), a subtype of ML that analyzes large amounts of data to improve the accuracy of creating concepts and accurately predicting pathologies (10), s currently one of the most widely used algorithms for medical purposes. ML has great potential in the medical field, including early prediction, rapid diagnosis, and risk assessment (11, 12).

AI plays an important role in thromboembolic diseases, especially in the early prediction and diagnosis of the disease (13). Early intervention with anticoagulants or other prophylaxis can reduce the incidence of PE and reduce patient mortality. A number of ML-based models have recently been developed to assess the risk of death from clinical disease, thereby enhancing clinical diagnosis and patient prognosis (14). Developing and validating ML models that accurately predict the risk of PE patients can help clinicians make informed decisions and improve patient survival. In this article, we summarize the current application of AI and ML in pulmonary embolism to elucidate the impact of AI in the medical field.

## 2 Basic concepts of AI and ML

AI, as a branch of computer science, explores and studies the nature of intelligent behavior and creates intelligent machines that respond in a similar way to human intelligence. These intelligent behaviors, including but not limited to learning, reasoning, problem solving, knowledge representation, planning, natural language processing, perception, pattern recognition, and creation, have become an umbrella term for technologies that mimic human “natural intelligence” or cognitive functions. ML is a subfield of AI that enables computers to derive knowledge and experience from data and use this knowledge and experience to make pattern recognition, prediction, and decision-making through the learning of computer systems and automated reasoning. In contrast to traditional statistical methods, which provide insights from observed inter-population differences based on collected clinical features, ML excels at revealing complex relationships between various features, thereby facilitating precise classification (15–17). Deep learning, on the other hand, is a subset of ML that represents an advanced approach to processing data using artificial neural networks with brain-like structures, with little or no artificial feature engineering, and is better at processing complex, high-dimensional data.

ML, the core discipline of AI, connects statistics and computer science to create predictive models capable of analyzing new data using large data sets (18), which stands out among several medical specialties and is the pathway to smart medicine (19, 20). Considering that clinicians need to evaluate large amounts of patient information to further guide clinical decision-making (21), it can be a daunting task for anyone. ML can create algorithms with comparable performance to human physicians (22), and when applied to medical images, algorithms are used to analyze and extract information from images generated by diagnostic imaging equipment, train algorithms to recognize patterns, identify relevant features, and perform specific tasks, such as diagnosing diseases, detecting abnormalities, or classifying medical conditions, and integrating and understanding relevant clinical data at scale, providing fast and accurate analytical tools to support medical professionals to assess risk, aid in diagnosis, It is excellent in judging prognosis and guiding personalized treatment, and is able to detect patterns or features that are not immediately visible to the naked eye, and is excellent in the processing of complex and large amounts of medical data (23, 24). ML has shown considerable potential in medical research and disease prediction (10, 25). For pulmonary embolism, ML models can help clinicians identify high-risk patients in the early stages of PE, enabling early intervention.

## 3 Application of AI in PE

### 3.1 Predictions

#### 3.1.1 PE

With the advent of AI as a predictive tool, some studies have applied AI to the prediction and screening of PE. The ML model performs better than the traditional risk scoring in PE risk stratification, and the new diagnostic model based on ML analysis can help overcome the limitations of the traditional score, improve the risk prediction performance, and accelerate the diagnosis of PE (26). A recent retrospective study analyzed the clinical data characteristics of 1,480 patients with suspected PE who were hospitalized in West China Hospital of Sichuan University from May 2015 to April 2020, used different MLs to make a PE prediction model, and evaluated their performance using the area under the receiver operating characteristic curve (AUC), and verified their predictive ability using SHapley Additive Interpretation (SHAP) values. The results showed an AUC of 0.776 (95% CI 0.774–0.778), suggesting that AI-based ML-based PE prediction models can help optimize early diagnosis and timely treatment strategies, thereby improving the prognosis of PE patients. At the same time, it provides literature support for the use of SHAP values to explain the contribution of ML in PE prediction. The convergence of engineering and medicine has shown excellent performance in predicting PE risk stratification (27). CTPA is the mainstay of clinical diagnosis of PE, but it is time-consuming, expensive, and in some cases unavailable, especially in primary care settings, and its simultaneous application may have adverse effects, particularly in patients with a history of kidney disease or allergies (28, 29). AI uses routinely collected patient healthcare data to derive the disease prognosis of patients with suspected PE, which can provide a reliable basis for patients’ care decisions. One study designed and evaluated a ML modeling approach called the PE Outcome Prediction Model (PERFORM) to predict PE imaging outcomes based on patient electronic medical record (EMR) data, including variables such as demographics, vital signs (absolute and change from baseline), diagnosis, medications, and laboratory test results, to provide patients with further CT imaging with a patient-specific risk score, improve detection rates, and reduce unnecessary CT examinations (30). To validate the performance of ML in predicting the risk of PE in high-risk inpatients with PE, Shen et al. retrospectively analyzed approximately 2 million adult hospitalized cases in the United States from 2011 to 2017, using demographics, vital signs, and laboratory tests of adult inpatients from 12 institutions to train the XGBoost model (31), and patient populations from 32 healthcare facilities performed external validation of the model, as measured by the area under the receiver operating characteristic curve (AUROC) Evaluating model performance, using backward elimination regression to determine the correlation between external validation set features and AUROC, the results showed that the external test AUROC ranged from 0.79 to 0.93 with a mean of 0.88, and the backward elimination regression determined a negative correlation between the percentage of PE positive visits and AUROC (*β* = −0.015, *p* < 0.001), indicating that this PE prediction model performed well in different external patient populations, demonstrating its potential as a clinical decision support tool (32).

#### 3.1.2 Other diseases combined with PE

PE itself may lead to pulmonary hypertension, cardiac insufficiency, respiratory failure and other serious consequences, and these pathological processes may be further exacerbated when complicated by other diseases, and the disease is often more complex, making diagnosis and treatment more difficult, and the prognosis is often poor, and delayed or missed diagnosis can be life-threatening. Therefore, the development of AI models to predict the risk of PE in other diseases and predict the risk of PE in other diseases plays an important role in improving the survival rate of patients and improving the quality of clinical decision-making.

Heart failure is a risk factor for predicting and stratifying mortality and risk stratification for PE, and short-term mortality is high in patients with PE (PE) and heart failure (33, 34). There is a need to predict the short-term mortality risk of patients with PE in heart failure in clinical practice, reduce death and disability through early detection and prevention, and improve patient outcomes. Liu et al. enrolled 472 patients with PE and heart failure, developed 6 ML models after feature selection, and externally validated patients in the eICU Cooperative Research Database (eICU-CRD) (35) by area under the curve (AUC), calibration curve, decision curve analysis (DCA), net weight classification improvement (NRI), and comprehensive discrimination improvement (IDI) to evaluate predictive performance, The Support Vector Machine model was found to perform the best prediction, with an AUC of 0.835, a higher degree of calibration, a wider risk threshold for clinical benefit (from 0 to 90%), and better than traditional mortality risk assessment systems such as the PE Severity Index and the Simplified PE Severity Index (34, 36, 37). Atrial fibrillation is one of the important factors contributing to the increase in morbidity and mortality worldwide, and its abnormal flow pattern can lead to the formation of blood clots in the atrium, which can break off and spread through the bloodstream, leading to arterial occlusion in various organs, such as PE (38). In order to predict the occurrence of composite thromboembolic events in elderly patients with atrial fibrillation, REN et al. collected data from 6,079 elderly inpatients admitted to the General Hospital of the Chinese People’s Liberation Army from January 2010 to June 2022, and randomly divided them into a training dataset (*n* = 4,225) and a validation dataset (*n* = 1,824) in a ratio of 7:3, and trained on the training dataset using four ML models (logistic regression, decision tree, random forest, XGBoost). The results showed that the random forest model showed good clinical validity, outperforming other models (ACC: 0.9144, SEN: 0.7725, SPE: 0.9489, AUC: 0.927, 95% CI: 0.9105–0.9443), showing the best predictive performance (39). Sun et al. conducted a retrospective observational study on data from 930 patients, developed five ML prediction models using data from the development cohort, selected the best model through five-fold cross-validation, and validated using an external validation cohort to assess the risk of PE in tuberculosis patients, and the results showed that the random forest model outperformed the other models with an AUC of 0.839 (95% CI 0.780–0.899) and maintained the highest average performance (AUC: 0.906 ± 0.041), indicating that random forests have significant advantages in predicting the risk of PE in TB patients compared to various ML models used to predict PE in TB patients, and demonstrating the application of AI in healthcare (40). Patients with autoimmune inflammatory rheumatic diseases have a high mortality rate after PE (41), and accurate stage prediction and risk assessment can improve survival. Hu et al. conducted a retrospective, multicenter study designed clinical data from 7,254 patients in Tongji Hospital from 2014 to 2022 to train an ML model using univariate logistic regression and minimal absolute contraction and selection operators to select clinical features, and the results showed that the ML-based model can accurately and conveniently predict the occurrence of PE in patients with autoimmune inflammatory rheumatism who are clinically suspected of PE (42). This study significantly improves the efficiency of predicting specific pathophysiological conditions, provides substantial help for doctors to finalize the diagnosis, and realizes the early and accurate diagnosis and risk prediction of PE in other diseases in clinical practice. It is of great significance in the field of prevention and treatment of PE to develop AI models that predict the risk of PE in other diseases and improve the early warning ability of patients with PE risk of other diseases.

### 3.2 Screening and diagnosis

PE is a serious medical emergency that requires prompt recognition and intervention, and early initiation of anticoagulation may improve outcomes for suspected PE (43). However, due to nonspecific symptoms associated with PE, less than 10% of patients evaluated are ultimately diagnosed with PE (44, 45). Missed diagnoses and misdiagnoses lead to delays in treatment and exacerbate disease progression (46–48). Therefore, the development and validation of natural language processing models to identify low-risk PE in real time and facilitate safe outpatient management is critical to reduce mortality from PE (49–51). In asymptomatic patients, radiologists may overlook most incidental PEs. The AI model can quickly analyze a large amount of imaging data, maintain sensitivity and specificity when interpreting suboptimal CTPA examinations, significantly enhance the detection of occasional PE, and significantly shorten the diagnosis time, which is crucial for patients with acute PE who need timely diagnosis and intervention, helping to shorten the time from symptom onset to diagnosis, and buying valuable treatment time for patients. AI algorithms have shown significantly higher sensitivity in detecting PE in routine scans compared to initial reports from radiologists, and the application of AI has significantly reduced the rate of missed diagnosis of incidental PE by radiologists (52). For example, one study showed that the implementation of AI algorithms significantly reduced the missed diagnosis rate from 50 to 7.1% compared to the absence of AI assistance, resulting in improved diagnostic accuracy (53). Langius-Wiffen et al. retrospectively analyzed the data of serial CTPA scans of 3,316 patients referred for suspected PE between February 24, 2018 and December 31, 2020, using a CE-marked and FDA-approved AI algorithm (54), and compared the output of the AI with the report of the attending radiologist, and the results showed that the sensitivity of the AI algorithm in detecting PE was significantly higher compared to the radiology report (96.8% vs. 91.6, *p* < 0.001). The specificity of AI was also significantly higher (99.9% vs. 99.7%, *p* = 0.035), and the implementation of AI-assisted reporting could reduce the number of missed (55). The development of AI methods with good diagnostic capabilities for PE can improve the performance and reproducibility of radiological diagnostics (56–58).

Ventilated/perfused single-photon emission computed tomography (V/Q-SPECT) is one of the most widely used imaging techniques for the diagnosis of PE, according to the 2019 guidelines for the diagnosis and management of PE developed in collaboration with the European Respiratory Society, however, V/Q-SPECT is invasive, expensive, and not as widely available as other imaging modalities such as CT, especially in resource-limited hospital settings (59). Li et al. selected potential perfusion-related features for integration into the model and found that the spatial distribution of features and the visualization of model output showed a high degree of agreement with lung function imaging, validating the feasibility of using quantitative texture analysis and data-driven ML pipelines to generate voxel-level lung perfusion surrogates between different institutions, providing a radiation-free, widely available alternative for functional lung imaging for the management of pulmonary vascular disease, and facilitating noninvasive screening of PE (60). AI-assisted workflows prioritize episodic PE with routine CT scans, and cancer patients have demonstrated high diagnostic accuracy (61). Wiklund et al. retrospectively analyzed the impact of AI algorithms on the detection rate of incidental PE in cancer-related patients, and showed that the reported prevalence of incidental PE was significantly higher and significantly shortened the reporting turnaround time and treatment time of patients with cancer-related incidental PE for a period of time after the implementation of AI (62).

Compared with larger embolism, small and easily overlooked PE has the characteristics of insidious symptoms, difficult diagnosis, and the need for active prevention and screening, which makes the detection of small PE often lead to misdiagnosis and missed diagnosis, therefore, in clinical work, doctors should maintain a high degree of vigilance, conduct detailed medical history and physical examination of patients suspected of PE, and timely diagnosis and treatment, and solve the detection problem of small PE is very important to improve the overall diagnostic results. In order to improve the identification and diagnosis of small and easily overlooked PE, Wu et al. collected the data of 142 CTPA examinations conducted at Tianjin Medical University General Hospital from January 2017 to October 2018 for a retrospective study, and developed a deep learning model SPE-YOLO, which showed 90.70% sensitivity and 86.45% accuracy in the data of the external validation set, showing strong diagnostic ability in identifying small PE, It provides clinicians with more accurate and efficient diagnostic tools (63). Yehuda et al. collected demographic, comorbidity, and drug data from 2,568 patients with PE and 52,598 control patients to build an accurate and informative ML model that would facilitate the early diagnosis of PE during patient hospitalization (64). Deep learning can improve detection performance and help clinicians complete diagnostic tasks (65–69).

### 3.3 Prognosis

The development and application of AI models is an important part of promoting the intelligent transformation of the medical industry, which will help improve the prognosis and quality of life of patients with PE. By analyzing the patient’s clinical data, imaging features, laboratory test results and other information, AI can predict the future development trend of the patient’s disease and the risk of possible complications, which can help clinicians formulate coping strategies in advance, guide subsequent treatment, and improve patient prognosis. ML models have been shown to accurately predict the 30-day mortality rate in critically ill PE patients, which can be further used to reduce the burden of ICU admissions, reduce mortality, and improve the quality of life of critically ill PE patients (70). Lian et al. conducted a retrospective analysis of the clinical data of 312 patients diagnosed with PE using CTPA in the Taicang Affiliated Hospital of Soochow University from 2016 to 2024 to evaluate the sensitivity, accuracy, specificity and AUC of four ML models (XGBoost, Random Forest, Logistic Regression and SVM) in predicting the prognosis of PE, and the results showed that the XGBoost model showed good performance, The accuracy was 0.882, the F1 score was 0.889, the precision was 0.917, the sensitivity was 0.863, the specificity was 0.905, and the AUC was 0.873. Future studies could combine these data to enhance the understanding of PE risk factors and improve predictive models for better clinical outcomes (71). Sadegh-Zadeh et al. utilized different oversampling techniques to improve the performance of various ML models, for early mortality prediction, and the results showed that the randomly oversampled RF model demonstrated superior performance across the 5 models evaluated, achieving higher accuracy and precision in predicting death grades, The study also highlighted the potential of ML to improve the accuracy of mortality prediction in patients with acute PE and provide a theoretical basis for clinical decision-making (72).

Anticoagulation is the mainstay of treatment for patients with PE (73). However, in clinical practice, some patients with PE may need to discontinue the drug early for a variety of reasons, including active bleeding, urgent need for surgery, contraindications to anticoagulation, etc. Identifying the likelihood of an increased risk of adverse outcomes before discontinuing anticoagulation can help improve patient outcomes. Mora et al. used data from the RIETE (74) registry to compare the prognostic capabilities of five ML models, including decision trees, K-nearest neighbors, support vector machines, ensembles, and neural networks (NN), to evaluate model performance by evaluating the model performance by measuring the test data for each model and the confusion matrix measure of the calibration plot to assess patients at increased risk of a compound of fatal PE or recurrent venous thromboembolism 30 days after discontinuation of the drug. The results showed that the ML-NN method was able to reliably predict fatal PE, sudden death, or recurrent VTE after premature cessation of anticoagulation, with an area under the ROC curve of 96 percent, an improvement of approximately 0.20 over traditional logistic regression, far exceeding the performance of traditional logistic regression models (75). Muñoz et al. extracted unstructured data from electronic health records of 9 hospitals in Spain between 2014 and 2018 using natural language processing (NLP) and ML-based EHRead@ techniques, performed clinical feature selection, and trained different predictive models to assess the risk of VTE recurrence within 6 months in cancer patients who received anticoagulation therapy at the time of initial VTE diagnosis. The findings can help clinicians identify high-risk patients and improve their clinical management (76).

## 4 Challenges and future of AI in PE

The development and implementation of AI in various clinical efforts across the medical field requires the inclusion of more clinical data in multiple centers and the minimization of possible bias due to unbalanced training, poor architecture design or selection, and uneven application of models, which can not only improve the reliability and accuracy of predictive models, but also avoid the influence of regional or technical factors and demographic characteristics on the model (77, 78). According to the FDA, despite tens of thousands of articles related to AI and computer-aided diagnosis (CAD) published over the past 20 years, as of July 30, 2023, only 692 market-liquidated AI medical algorithms are available in the United States (79). When clinical data is only partially available and laboratory test results are still unavailable, the diagnosis needs to be made immediately after the patient’s emergency department presentation. In a study conducted by Cheik et al., software utilizing AI for image interpretation demonstrated the ability to identify 219 suspected PEs, of which 176 were confirmed to be true PE. The AI system had the highest sensitivity and negative predictive value (NPV) of 92.6 and 98.6%, respectively, outpacing radiologists who achieved 90% sensitivity and 98.1% NPV. Meanwhile, radiologists performed well in specificity and positive predictive value, with a specificity of 99.1% and a positive predictive value of 95%, compared to a specificity of 95.8% and a positive predictive value of 80.4% for AI. These results highlight the potential of AI to enhance PE detection, while also revealing the excellent performance of radiologists in reducing false positives (80). In order to address the challenge of bias in algorithm development brought about by population differences within the healthcare system, a bias-free symptom prediction framework was proposed using the state-of-the-art PE detection backbone and large-scale clinical language model, which achieved a higher survival correlation than the clinical assessment index, reduced the inequity of medical implementation leading to PE prognosis and other diseases, and improved the fairness and accuracy of medical AI systems (81). At the same time, the integration of ML into clinical practice introduces key ethical considerations, including issues of liability for medical errors, healthcare professionals’ understanding of how these algorithms generate predictions, and concerns about ethics, safety, and patient data control (82, 83).

AI has played an important role in the prediction, diagnosis, and prognosis assessment of PE, and with the popularization of PE prediction models, attempts have been made to construct optimal PE prediction models based on cause classification to maximize the clinical outcomes of PE patients (84, 85). Future research should continue to evaluate the feasibility and cost-effectiveness of clinically relevant AI tools, requiring further research and interdisciplinary collaboration to engage a variety of stakeholders in AI development and deepen insights into potential biases and ethical considerations. Select, support, and fund more highly focused AI projects that address challenges encountered during development and implementation, and further explore the impact of AI on patient outcomes.

## 5 Conclusion

In the medical field, AI has many applications, followed by significant advances in a relatively short period of time (86). ML is one of the fastest and most convenient methods to detect diseases through AI techniques (87, 88). The synergy between ML and medicine has transformed disease prevention and treatment (89). Given the sheer volume and complexity of clinical data generated today, diagnostic algorithms based solely on physician clinical experience or traditional statistical methods often fail to deliver superior performance. ML, as part of artificial intelligence, has strong learning capabilities and predictive performance, the ability to process large cubes, identify patterns and correlations that may not be apparent using traditional statistical methods, and objectively analyze large amounts of data to predict clinical outcomes, which can improve diagnostic accuracy and predict complex medical conditions (90, 91). PE (PE) is a major health problem, ranking third among cardiovascular diseases (92). By analyzing medical image data, AI-based applications can effectively extract important features from images, enhance their utility in medical image analysis, identify potential missed PE, improve the accuracy and efficiency of PE diagnosis, and improve patient outcomes through timely intervention (93–96). Therefore, this article summarizes the application performance of AI in the optimization of clinical decision-making of PE, which has significant advantages in the early prediction of PE, timely screening and diagnosis, and improved prognosis. The development of predictive models with strong generalization capabilities to assess the risk of PE in patients using ML algorithms has great potential for development (97). However, rigorous implementation and validation are essential to ensure the safety and efficacy of these techniques in clinical practice.
